# Practical Tips for Developing Your Staff

**DOI:** 10.5195/jmla.2017.271

**Published:** 2017-07-01

**Authors:** Martha Earl

This work, part of the Practical Tips for Library and Information Professionals series, was developed to provide creative tips-based approaches and best-practice ideas supporting innovation and adaptation in the constantly changing library environment. Grounded in theory, but not overly theoretical, this guide will assist librarians in seeking new ideas and inspiration to achieve continuous professional development (CPD). Acronyms abound, but since this book originates from the United Kingdom, the jargon differs. Authors have conveniently provided a “List of Abbreviations.”

Staff in any organization are the most valuable asset. Ensuring the continued development of that staff to not only perform optimally, but also to thrive can be a challenge. Perhaps even more difficult than encouraging adaptation to change is encouraging individuals to challenge themselves to move forward in their careers or job satisfaction.

In this volume, the manager can find advice on a variety of topics, such as enabling others to plan, evaluate, and reflect on their personal development; appraising and setting goals; linking personal objectives to organizational objectives; managing performance; locating funding to attend and host events; planning formal development activities such as courses and conferences; accessing informal activities; using social media as a development tool; understanding the role of professional associations and networks; and mentoring, shadowing, coaching, and other modes of networking.

A significant strength of this volume lies in its organization. For each tip, the authors provide an “Overview” of the tip or activity followed by other information including “Best for,” the context where this tip is best applied; “More,” examples of how the tip or activity can be adapted; “Watch out,” practical advice on pitfalls that can happen when using the outlined approach; and “References,” the research that supports the practice.

Section 1, “Theories,” includes a particularly valuable set of efficiently written descriptions of major theories of human behavior, learning, and development along with an introduction as to why familiarity with these theories can ground the manager or leader interested in developing staff. These nine theories can be quickly scanned with the option for more reading, if so desired. Section 2, “Infrastructure,” provides an overview of basic organizational structures that are necessary for learning and development to occur. These twenty-two topics, from “Work-force Planning” to “Effective Hand-over,” include tips on recruitment, interviewing, performance appraisal, team building, and exit procedures. Section 3, “Activities and Tools,” succinctly covers a fairly comprehensive list of sixty-eight ideas and methods to aid individuals in meeting a variety of career development goals. Listed in alphabetical order, these activities can predominantly be incorporated into a person’s work day.

Provided tips can enhance such everyday activities as attending or running meetings, training staff, writing reports, and presenting at conferences. The essence is that career development is a continuous process, not necessarily requiring protected time, though such topics as reflective writing and social media are also addressed. CPD is the heart of a successful and satisfying career.

The authors did not intend the book to be read in a linear fashion but to serve as a reference tool. Each tip provides an overview and details, guidance on timing, and issues to consider when employing the technique. The work is a pathfinder for continuous professional development, regard-less of available money and time. The authors provide numerous figures and tables to support the text, with a list of figures and tables. The index is cross-referenced. References include both classic works and current resources.

Tracey Pratchett, currently knowledge and library services manager at Lancashire Teaching Hospitals, served as the joint project lead for the Making Alignment a Priority (MAP) toolkit. Gil Young, the National Health Service Library and Knowledge Services Workforce development manager for the Health Care Libraries Unit North, also was noted as a Chartered Institute of Library and Information Professionals Fellow and Mentor of the Year. Both authors have additional experience in academic and public libraries.

This book is recommended for all library types as part of a librarian’s library. Although the tips and activities could be utilized by professionals in any field, the examples are specific to libraries. One could safely place this title in general collections for other managers to use or for those seeking a reference for personal professional development.

